# A Splicing-Dependent Transcriptional Checkpoint Associated with Prespliceosome Formation

**DOI:** 10.1016/j.molcel.2014.01.017

**Published:** 2014-03-06

**Authors:** Keerthi T. Chathoth, J. David Barrass, Shaun Webb, Jean D. Beggs

**Affiliations:** 1 Wellcome Trust Centre for Cell Biology, University of Edinburgh, King’s Buildings, Mayfield Road, Edinburgh EH9 3JR, UK

## Abstract

There is good evidence for functional interactions between splicing and transcription in eukaryotes, but how and why these processes are coupled remain unknown. Prp5 protein (Prp5p) is an RNA-stimulated adenosine triphosphatase (ATPase) required for prespliceosome formation in yeast. We demonstrate through in vivo RNA labeling that, in addition to a splicing defect, the *prp5-1* mutation causes a defect in the transcription of intron-containing genes. We present chromatin immunoprecipitation evidence for a transcriptional elongation defect in which RNA polymerase that is phosphorylated at Ser5 of the largest subunit’s heptad repeat accumulates over introns and that this defect requires Cus2 protein. A similar accumulation of polymerase was observed when prespliceosome formation was blocked by a mutation in U2 snRNA. These results indicate the existence of a transcriptional elongation checkpoint that is associated with prespliceosome formation during cotranscriptional spliceosome assembly. We propose a role for Cus2p as a potential checkpoint factor in transcription.

## Highlights

• Transcriptional elongation is inhibited when prespliceosome formation is blocked
• The defect is characterized by RNA polymerase accumulation on introns
• This checkpoint can be triggered by mutations in either *PRP5* or U2 snRNA
• The U2-associated Cus2 protein is a candidate checkpoint factor

## Graphical Abstract

**Figure:**
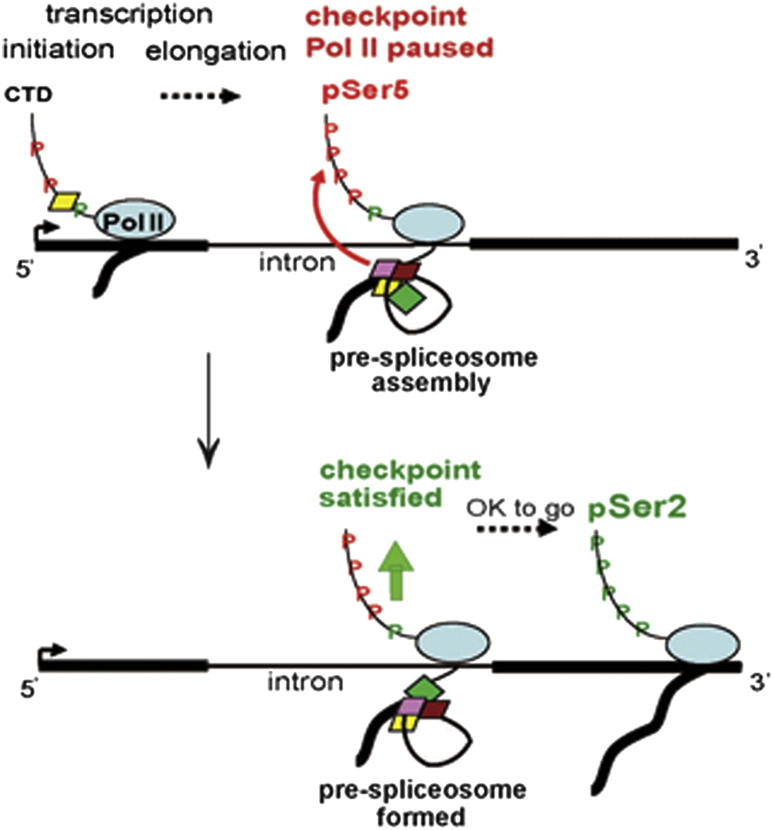

## Teaser

In eukaryotes, transcription and splicing are functionally linked, but how or why this happens is unknown. Chathoth et al. show that, in budding yeast, these processes are coupled by one or more transcriptional checkpoints associated with surveillance of the splicing machinery as it assembles cotranscriptionally on nascent transcripts.

## Introduction

Transcription and precursor mRNA (pre-mRNA) processing, initially considered to be independent processes, are now known to be intimately linked. There is appreciable evidence that 5′ capping and 3′ end cleavage and polyadenylation generally occur cotranscriptionally, and splicing often does so too, with the carboxy-terminal domain (CTD) of the largest subunit of RNA polymerase II (Pol II) playing a significant role in coupling transcription to these processing events (McCracken et al., 1997; Bentley, 2005; Moore and Proudfoot, 2009; de Almeida et al., 2010). The CTD contains multiple highly conserved heptapeptide repeats that undergo cycles of posttranslational modifications, with the best studied being phosphorylation of serines at positions 2 and 5 of the repeat unit. The phosphorylation status of the CTD modulates its interactions with RNA processing factors at different stages of transcription (Egloff and Murphy, 2008; Buratowski, 2009; David and Manley, 2011). In *Saccharomyces cerevisiae*, the RNA capping enzymes are recruited cotranscriptionally via phosphorylated Ser5 (pSer5) of the CTD (Cho et al., 1997; Schroeder et al., 2000), and 3′ end cleavage and polyadenylation factors are recruited through phosphorylated Ser2 (pSer2) in vivo (Ahn et al., 2004) and in vitro (Hirose and Manley, 1998).

Certain splicing factors, including some members of the serine- and arginine-rich (SR) family of proteins (U2AF, SC35), and some small nuclear ribonucleoprotein particles (snRNPs) were also shown to have links with Pol II (reviewed in Kornblihtt et al., 2004; Lin et al., 2008; Ji et al., 2013). Transcriptional elongation complexes were reported to contain splicing proteins in addition to elongation factors (Kameoka et al., 2004). Introns were also observed to enhance transcription (Furger et al., 2002). In yeast, snRNPs were shown to be recruited sequentially to the nascent transcript, indicative of cotranscriptional spliceosome assembly (Görnemann et al., 2005; Lacadie and Rosbash, 2005; Tardiff and Rosbash, 2006).

Coupling of splicing and transcription could permit their coregulation. It was reported that different promoters, initiation rates, and elongation rates of transcription can affect the outcome of splicing (Cramer et al., 1999; Das et al., 2007; Witten and Ule, 2011). Conversely, splicing can influence transcriptional elongation (Lin et al., 2008; Singh and Padgett, 2009) and initiation rates (Damgaard et al., 2008). A genome-wide analysis of nascent RNA in yeast reported polymerase pausing on terminal exons encoding cotranscriptionally spliced transcripts (Carrillo Oesterreich et al., 2010). In addition, a high-resolution kinetic study of a reporter gene in yeast revealed splicing-dependent RNA polymerase pausing near the 3′ end of the intron, which was proposed to correspond to a transcriptional checkpoint (Alexander et al., 2010a).

Spliceosome assembly is a multistep process in which the U1, U2, U4/U6, and U5 snRNPs and non-snRNP splicing factors interact with the pre-mRNA and with each other, defining the intron splice sites and the branchpoint (BP) (reviewed in Wahl et al., 2009). The 5′ splice site (5′SS) is recognized by the U1 snRNP and the BP by the SF1/BBP and U2AF proteins (Msl5 and Mud2 in yeast) that form the commitment complex. The U2 snRNP then associates with the BP, leading to formation of the prespliceosome, or complex A. Complex A is converted to complex B by addition of the U4/U6 and U5 snRNPs in the form of a preassembled tri-snRNP particle. A major reorganization occurs that displaces the U1 and U4 snRNPs, accompanied by the addition of the multiprotein nineteen complex. A further reorganization is required to activate the spliceosome for the first catalytic step of splicing. As a consequence of the first step, complex C is formed. This is reorganized again to perform the second reaction. Finally, the spliceosome is actively dissociated, and the products of splicing are released.

Eight RNA-stimulated adenosine triphosphatases (ATPases) are required to promote the various conformational rearrangements during the cycle of spliceosome assembly, catalysis, and disassembly (Cordin and Beggs, 2013). Among these, Prp5 protein (Prp5p) plays a role in prespliceosome assembly and was proposed as a fidelity factor that proofreads interaction between U2 snRNA and BP (Xu and Query, 2007). Studies carried out in the Ares lab showed that Prp5p, together with the U2 snRNP-associated protein, Cus2p, promotes a conformational change in U2 snRNA from stem IIc to stem IIa (Perriman et al., 2003; Perriman and Ares, 2007). This allows U2 snRNA to interact with the BP sequence in the intron. Cus2p is then displaced from the complex as a consequence of ATP hydrolysis by Prp5p, resulting in prespliceosome formation (Perriman et al., 2003; Perriman and Ares, 2000; Yan et al., 1998). When Cus2p is absent, U2 snRNA adopts the stem IIa form more readily, and the ATP-dependent activity of Prp5p is less necessary (Perriman et al., 2003). The heat-sensitive *prp5-1* mutant is defective in formation of prespliceosomes when shifted to the nonpermissive temperature, and heat-treated splicing extract from *prp5-1* cells accumulates a complex containing Prp5p, U1, and U2 snRNP proteins (Ruby et al., 1993). The *prp5-1* mutation lies in motif I, which is conserved in DEAD-box proteins. This motif is generally important for ATP binding by DEAD-box proteins, and the *prp5-1* mutation is thought to cause a defect in the ATP-dependent remodeling of U2 snRNA, although loss of ATPase activity has not been demonstrated directly (Xu and Query, 2007). Certain *prp5* mutations that reduce ATPase activity improve the splicing of introns with suboptimal BP sequences, although the *prp5-1* allele apparently does not have this property (Xu and Query, 2007).

Here, we present chromatin immunoprecipitation (ChIP) evidence for a transcriptional elongation defect in which Pol II that is mainly phosphorylated at Ser5 of the CTD accumulates over introns in a *prp5-1* strain at the restrictive temperature. ChIP sequencing (ChIP-seq) analysis with *prp5-1* cells reveals that Pol II is enriched on introns genome wide. Furthermore, we show that Pol II stalling in *prp5-1* cells is mediated through the U2 snRNP-associated Cus2p and that reduced nascent transcript production from intron-containing genes in the *prp5-1* strain is suppressed by deletion of *CUS2*. A similar accumulation of Pol II is observed when prespliceosome formation is blocked by a mutation in *SNR20* that causes hyperstabilization of the stem IIc form of U2 snRNA. We propose the existence of a transcriptional elongation checkpoint that is associated with prespliceosome formation during cotranscriptional spliceosome assembly and that is also triggered by a branchpoint mutation in the intron. These observations suggest a role for Cus2p as a potential checkpoint factor in transcription.

## Results

### Hyperphosphorylated RNA Pol II Accumulates over Introns in *prp5-1* Cells

**Figure 1:**
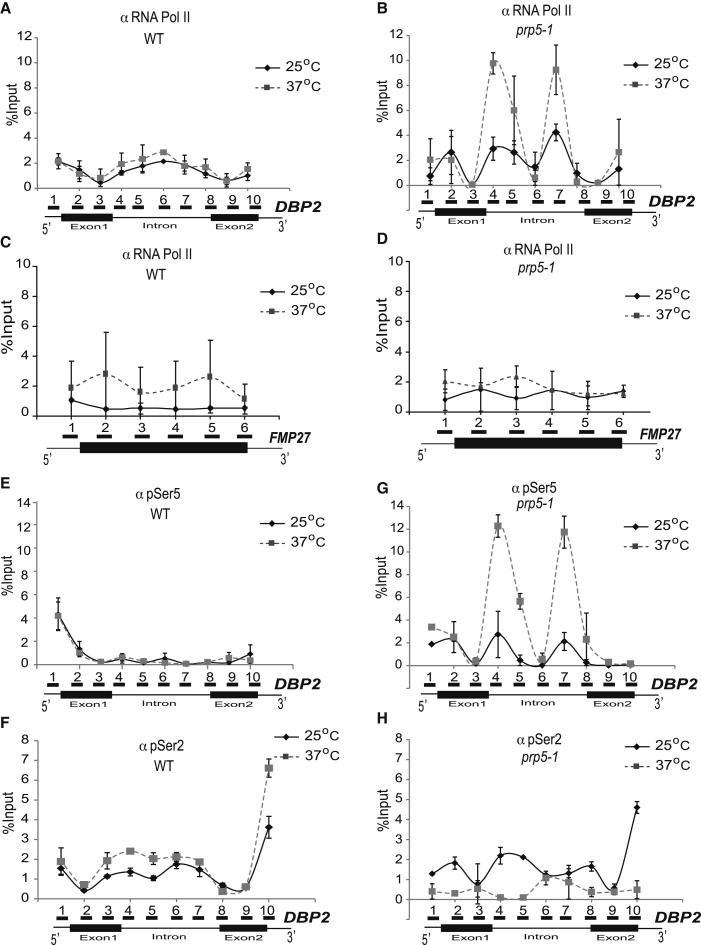
ChIP-qPCR Analysis Shows Accumulation of pSer5 Pol II over Introns in *prp5-1* Cells (A–H) Cells grown to mid-log phase were shifted from 25°C to 37°C. Samples were taken and formaldehyde crosslinked before the shift (25°C) and after 30 min at 37°C. ChIP was performed using antibodies 4F8 (total RNA Pol II), 3E8 (pSer5), or 3E10 (pSer2) as indicated, followed by qPCR analysis. Solid and dashed lines denote 25°C and 37°C, respectively. Positions of amplicons are shown below each graph. ChIP was performed in WT cells using 4F8 (total RNA Pol II) (A), *prp5-1* cells using 4F8 (B), WT cells using 4F8 (C), *prp5-1* cells using 4F8 (D), WT cells using 3E8 (pSer5) (E), WT cells using 3E10 (pSer2) (F), *prp5-1* cells using 3E8 (G), and *prp5-1* cells using 3E10 (H), followed by qPCR analysis. (A), (B), and (E)–(H) used *DBP2* (intron-containing), and (C) and (D) used *FMP27* (intronless). Error bars indicate SEM from two independent experiments, each assayed in duplicate. Figure S1 shows a time course of Pol II ChIP on *DBP2* in *prp5-1* cells during incubation at 37°C, plus similar analyses with *ACT1* and *ASC1*.

To test the proposal that a defect in Prp5p affects transcription and might invoke a splicing-related transcriptional checkpoint, we performed ChIP to examine Pol II occupancy along the length of several intron-containing genes in wild-type (WT) and *prp5-1* mutant cells. Antibodies (4F8; Chapman et al., 2007) that immunoprecipitate all forms (modified and unmodified) of Pol II produced a relatively uniform signal across the long *DBP2* gene in WT cells at 25°C and 37°C (Figure 1A). Strikingly, shifting *prp5-1* mutant cells to the nonpermissive temperature for 30 min resulted in an accumulation of Pol II over the intron (Figures 1B and S1A available online). However, this was not observed with the temperature-sensitive mutant *prp8-R1573K* that affects a different stage of splicing (Figure S1B; Schneider et al., 2004), indicating that this is not a general consequence of defective splicing. Pol II also accumulated on the intron-containing *ACT1* and *ASC1* genes in the *prp5-1* mutant (data not shown, but see Figure S1), but not on the intronless *FMP27* gene (Figures 1C and 1D).

We then performed ChIP analysis with antibodies specific for CTD with pSer5 or pSer2 (Chapman et al., 2007). This showed Pol II with pSer5 elevated at the 5′ end and Pol II with pSer2 higher toward the 3′ end of the *DBP2* gene in WT cells at both 25°C and 37°C (Figures 1E and 1F), as expected (Komarnitsky et al., 2000). Analysis of *prp5-1* cells revealed that the Pol II that accumulated over the *DBP2* intron at 37°C was strongly phosphorylated on Ser5, but not on Ser2, of the CTD (Figures 1G and 1H). Similar results were obtained with the intron-containing *ACT1* and *ASC1* genes (Figures S1C–S1J). The unusual accumulation of Pol II with pSer5 within the gene body in *prp5-1* cells indicates a possible transcriptional elongation defect. The intronless *FMP27* gene did not show this effect (Figure 1 and data not shown).

### Pol II Accumulates over Many Intron-Containing Genes in *prp5-1* Cells

**Figure 2:**
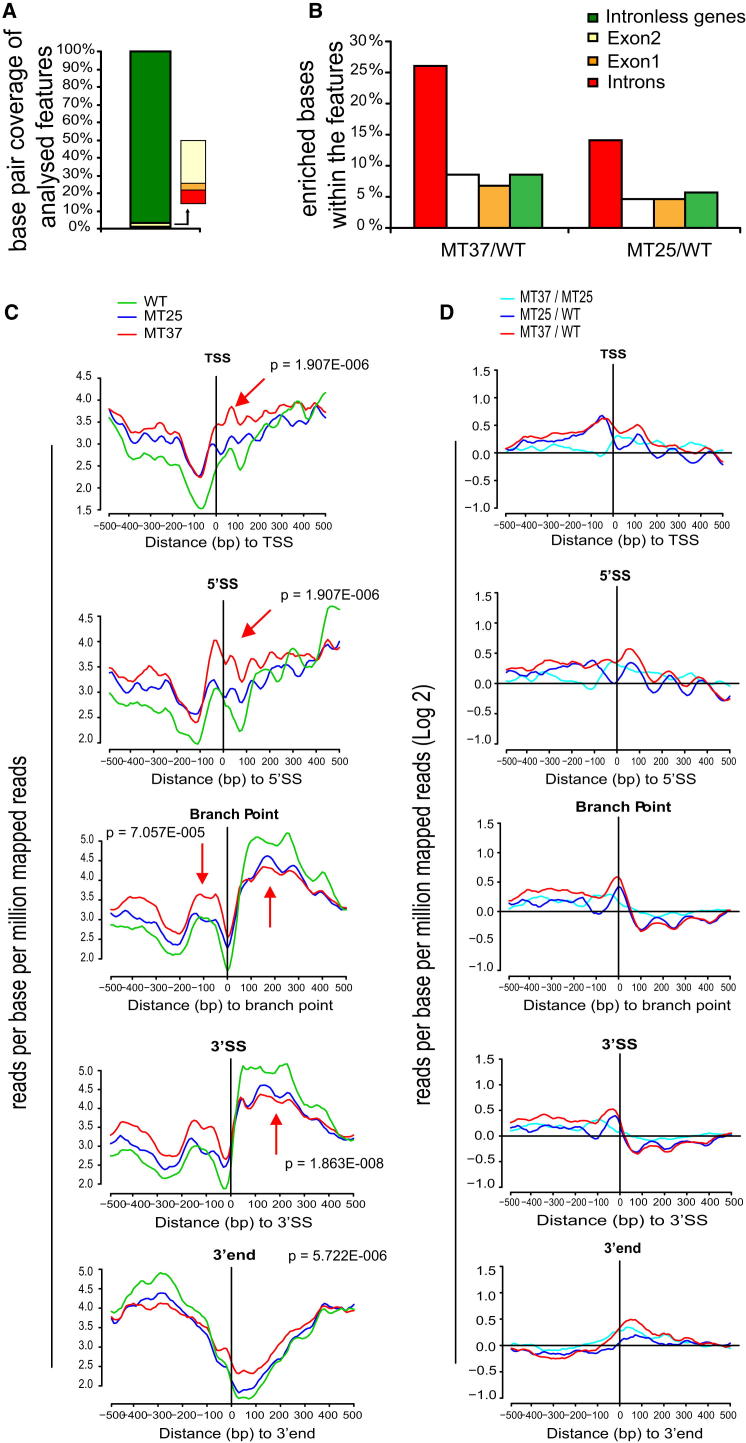
ChIP-Seq Analysis Shows Pol II Enrichment over Introns Genome Wide in *prp5-1* Cells (A) Bar chart displaying base pair coverage of regions as a percentage of the analyzed features: introns (0.72%), exon1 (0.36%), exon2 (2.27%), and intronless genes (96.64%). The relative values for introns, exon1, and exon2 are magnified alongside to facilitate comparison. (B) Bar chart showing the proportion of significantly enriched bases (>2-fold) on introns compared to other genome features. The data are presented for *prp5-1* (MT)/WT at 37°C and 25°C. (C) Proximity plot analysis for the number of base pair reads on intron-containing genes positioned with respect to 5′SS, BP, and 3′SS (n = 247) and TSS and 3′ end (n = 206). Green line, WT; blue line, MT25; red line, MT37. (D) Similar analysis performed as ratios: MT37/WT (red), MT25/WT (blue), and MT37/MT25 (turquoise) as indicated; p values are also indicated (see Experimental Procedures and Table S1 for details). See Figure S2 for examples of individual genes and other comparative analyses.

We next used ChIP-seq to investigate the effect of *prp5-1* on Pol II genome wide. Pol II data for *prp5-1* at 37°C and 25°C are compared to WT at 25°C (MT37/WT, MT25/WT). Looking genome wide at sequences that show at least 2-fold (>2) enrichment of Pol II, we calculated the proportion of enrichment over introns, exons, and intronless genes based on base pair coverage of each of these features in the genome (Figure 2A). In *S. cerevisiae*, only 5% of Pol II transcribed genes are intron containing, with all intron sequences corresponding to less than 1% of the genome. Considering first the intron sequences, 26% (of the base pairs) are enriched >2-fold in the mutant at 37°C (Figure 2B, MT37/WT). In contrast, exon sequences and sequences from intronless genes are much less enriched (8% of bp in exon1, 7% in exon2, and 8% in intronless genes) (Figure 2B). Thus, introns are strongly overrepresented as sites of Pol II accumulation in the mutant at 37°C. Out of 265 intron-containing genes analyzed, 209 were found to contain regions that were enriched at least 2-fold in the mutant at 37°C compared to WT (Table S1, Figure S2). Intron sequences are also enriched with Pol II in the mutant at 25°C relative to WT (MT25/WT), but to a lesser extent. Therefore, Pol II accumulates on introns in the *prp5-1* mutant even at the permissive temperature, but more so at the restrictive temperature (see also MT37/MT25 in Figure S2). This is compatible with Pol II pausing or stalling on introns in *prp5-1* cells.

In order to investigate the possibility that Pol II accumulates at recognized features in intron-containing genes, we plotted the number of reads per base relative to the transcription start site (TSS), 5′SS, BP, 3′ splice site (3′ SS), or 3′ end of the RNA for all intron-containing genes. First, this shows that for WT cells there is a drop in the number of reads immediately upstream of the TSS, followed by increased reads between the TSS and the 5′SS (Figure 2C, green line). There is a further increase in reads immediately upstream of the BP, followed by a pronounced dip and then a much greater increase immediately downstream of the 3′SS. As expected, the number of reads falls at the position where transcript 3′ ends map. These gene-averaged plots indicate that Pol II tends to accumulate upstream of BPs and piles up at the 5′ end of exon2 in the WT strain. Comparing the results for the mutant and WT strains, Pol II accumulates more in the *prp5-1* mutant around the 5′SS and prior to the BP, especially at 37°C (Figures 2C and 2D, red lines; p values are indicated). However, there is a transition point near the 3′SS, beyond which there are fewer reads for the mutant. This is compatible with a transcriptional elongation defect in the *prp5-1* cells apparently caused by Pol II accumulation over introns.

The results for individual genes show some heterogeneity in the pattern of Pol II enrichment in mutant versus WT, but there is generally an enrichment over introns, especially near the 5′ end and upstream of the BP (e.g., *DBP2* and *RPL42A*; Figure S2A). On intronless genes, the enrichment profile is strikingly different, with Pol II more uniformly distributed along the genes (e.g., *MPS3* and *FMP27*; Figure S2A, note the different scales). Also, the *prp5-1* strain shows greater Pol II enrichment and does so on more intron-containing genes at 37°C (209 genes) than at 25°C (176 genes) (Figures S2B–S2D). Most of the genes whose RNA splicing was previously reported to be reduced due to the *prp5-1* mutation, based on microarray analysis (Pleiss et al., 2007), are found among the Pol II enriched genes (Table S1).

Scaling (binning) the reads relative to a point 100 bp upstream of the 5′SS, the 5′SS itself, 3′SS, and 3′ end shows enrichment at the 5′ ends of genes in mutant strains at both temperatures compared to WT (Figures S2B [MT37/WT] and S2D [MT25/WT]), whereas the additional reads in the mutant at 37°C map predominantly over the introns (Figure S2C [MT37/MT25] and Table S1).

### *cus2Δ* Suppresses Pol II Stalling in *prp5-1* Cells

**Figure 3:**
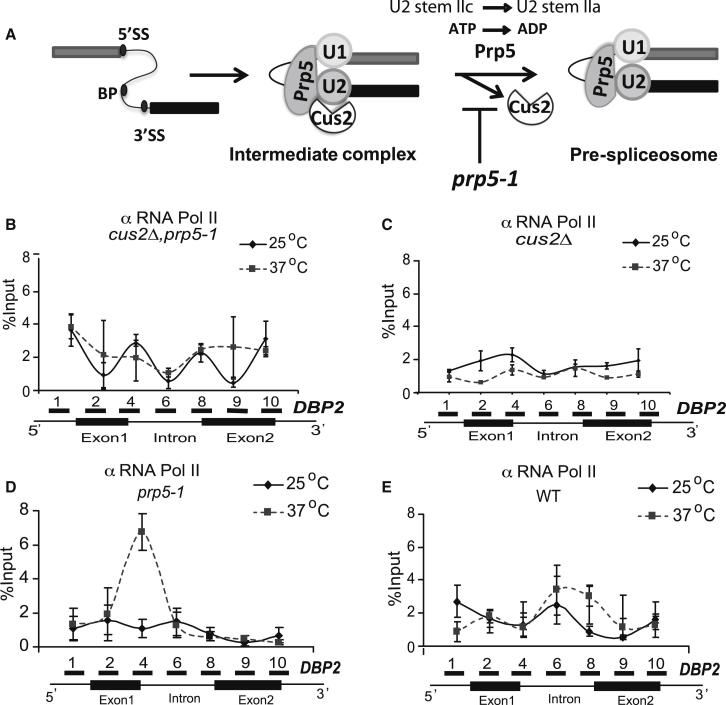
*cus2Δ* Abolishes Pol II Accumulation and Restores Transcription in *prp5-1* Cells (A) Schematic representation of Prp5p and Cus2p in prespliceosome formation. Prp5p joins U1 snRNP bound to the 5′SS (Kosowski et al., 2009), followed by U2 snRNP and associated protein Cus2p. The U2 snRNP is rearranged to form the prespliceosome complex after passing through an intermediate complex, where Cus2p is displaced and U2 stem IIc is converted to stem IIa upon ATP hydrolysis by Prp5p. In the mutant, Prp5-1p is present in the commitment/intermediate complex (Ruby et al., 1993; Perriman and Ares, 2010), but the *prp5-1* defect inhibits prespliceosome formation (Perriman et al., 2003). (B–E) ChIP-qPCR analysis of total Pol II (4F8 antibodies) at 25°C, or after 30 min shift to 37°C, on *DBP2* in *cus2Δprp5-1* (B), *cus2Δ* (C), *prp5-1* (D), and WT (E) cells, respectively. Positions of the amplicons are indicated on the x axis (a subset of primers from Figure 1 is used here). Solid and dashed lines denote 25°C and 37°C, respectively. Error bars represent SE from two independent experiments, each assayed in duplicate. See Figure S3 for a similar analysis of *ASC1* and for analysis of phosphorylated Pol II on *DBP2*.

Prior to prespliceosome formation, Prp5p remodels the U2 snRNA from the stem IIc to the stem IIa conformation, hydrolyses ATP, and displaces Cus2p from the complex. This facilitates U2 snRNA interaction with the intron branchpoint (Perriman et al., 2003). At the restrictive temperature, the mutant Prp5-1 protein fails to displace Cus2p from the complex (Perriman et al., 2003), blocking prespliceosome formation (illustrated in Figure 3A).

As the requirement for the ATP-dependent activity of Prp5p is reduced when Cus2p is absent, we investigated whether the presence of Cus2p affects Pol II behavior. *CUS2* is not essential for cell viability, and its absence does not cause any detectable splicing defect or cell growth defect (Yan et al., 1998). Therefore, *CUS2* was deleted from the *prp5-1* strain and from the isogenic WT strain, and ChIP-qPCR analysis was performed at 25°C and 37°C. Unlike *prp5-1*, but like WT, the *cus2Δ, prp5-1* double mutant and the *cus2Δ* cells exhibited a similar Pol II distribution at both temperatures, with no Pol II accumulation on either *DBP2* or *ASC1* (Figures 3B–3E and S3A–S3F). Reintroducing the *CUS2* gene on a plasmid restored Pol II accumulation in the double mutant at 37°C (Figure S3G), confirming that the peak of Pol II signal detected in *prp5-1* cells at 37°C was *CUS2* dependent. Therefore, the presence of Cus2p is required for Pol II accumulation at 37°C in *prp5-1* cells.

### *prp5-1* Causes a Transcription Defect, and *cus2Δ* Restores Transcription, but Not Splicing, in *prp5-1* Cells

**Figure 4:**
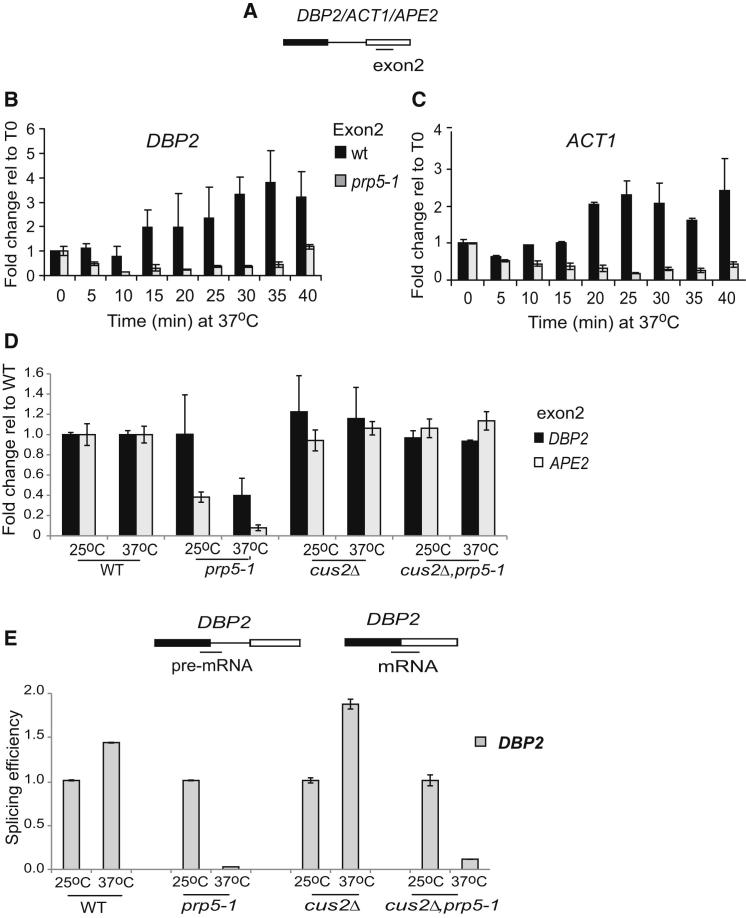
Intron-Containing Genes Exhibit a Transcription Defect in *prp5-1* Cells (A–C) Cells were grown at 25°C (permissive) and shifted to 37°C (nonpermissive), and total RNA was isolated at 5 min intervals up to 40 min. RT-qPCR results are plotted for 37°C relative to 25°C. Diagram illustrating RT-qPCR analysis of exon2 (A). Exon2 of *DBP2* (B) and *ACT1* (C) genes in WT and *prp5-1* showing reduced levels in the mutant at 37°C. (D and E) Cells bearing *FUI* plasmid were grown to mid-log phase in Ura dropout medium, and 4TU labeling was performed for 2 min at 25°C (permissive) or after a 30 min shift to 37°C (nonpermissive). Affinity-selected nascent RNA was analyzed by RT-qPCR on exon2 and around the 5′SS and splice junction. The diagrams show the positions of the amplicons generated. Levels of exon2 from intron-containing genes *DBP2* (black bar), *APE2* (white bar) in *prp5-1*, *cus2Δ,prp5-1*, and *cus2Δ* cells, presented relative to WT at different temperatures (D). Splicing efficiency measured as the ratio of mRNA/(mRNA + pre-mRNA) at 37°C relative to 25°C for *DBP2* in WT, *prp5-1*, *cus2Δ prp5-1*, and *cus2Δ*, showing decreased splicing efficiency in *prp5-1* and *cus2Δprp5-1* at 37°C (E). In all panels, bars indicate SEM from two independent experiments, each assayed by qPCR in triplicate. Results for intronless genes *FMP27* and *DAL80* and for effects on splicing are shown in Figure S4. As a measure of the effectiveness of the affinity purification of nascent RNA away from preexisting RNA, Figure S4I shows the PCR values for *DBP2* as fold over background (the same analysis without 4TU).

To investigate whether the Pol II accumulation observed in *prp5-1* corresponds to a transcriptional elongation defect, we first measured the effect of *prp5-1* on the levels of several intron-containing or intronless transcripts by reverse transcription and real-time quantitative PCR (real-time qPCR; Figure 4A) during a time course after shifting cultures from 25°C (permissive) to 37°C (restrictive). For the intron-containing genes, exon2 was measured as a representative of all versions of each transcript. After a short delay due to the heat treatment, the levels of *DBP2* and *ACT1* transcripts increased at 37°C in WT cells, but not in *prp5-1* cells (Figures 4B and 4C). In comparison, the levels of the intronless *FMP27* and *DAL80* transcripts fluctuated transiently after shifting to the elevated temperature but, overall, were not significantly affected by the *prp5-1* mutation (Figures S4B and S4C). RT-qPCR analysis showed accumulation of unspliced pre-mRNA and depletion of mRNA in the *prp5-1* cells at 37°C due to the splicing defect, as expected (Figures S4D–S4G). These experiments also show that the transient effect of heat treatment on transcription in WT cells was over by 15–20 min (Figures 4 and S4 and data not shown).

The analysis of total RNA, as above, is complicated by the presence of RNA that existed before the heat treatment as well as the effect of RNA turnover during the heat treatment. Therefore, we next measured the incorporation of 4-thio-uracil (4TU) into newly synthesized RNA during a 2 min period in *prp5-1* mutant and WT cells grown at 25°C, or after 30 min at 37°C. After rapid harvesting of the cells and extraction of RNA, the nascent, 4TU-labeled transcripts were affinity purified and analyzed by RT-qPCR. This showed that for the intron-containing *DBP2* and *APE2* genes, the amount of RNA produced at 37°C in the 2 min labeling period was greatly reduced in the *prp5-1* mutant compared to WT, especially for *APE2*, for which less RNA was produced even at 25°C (Figure 4D). In contrast, *prp5-1* did not reduce the transcription of the intronless *ALG9* and *FMP27* genes during the brief period of labeling (Figure S4H).

Strikingly, RT-qPCR analysis of nascent *DBP2* and *APE2* transcripts in *cus2Δ* and *cus2Δ,prp5-1* cells showed similar levels of exon2 at 25°C and 37°C compared to those of WT (Figure 4D), showing that the absence of Cus2p in the double mutant suppresses the RNA accumulation defect caused by the *prp5-*1 mutation. However, as previously reported (Perriman et al., 2003), *cus2Δ* did not bypass the role of Prp5p in splicing, as *cus2Δprp5-1* cells have a splicing defect at 37°C similar to that of *prp5-1* (Figure 4E). Overall, it seems that the *prp5-1* mutation reduces accumulation of nascent RNA from the tested intron-containing genes in a Cus2-dependent manner and not simply as a consequence of the splicing defect.

### The *snr20-G53A* Mutation Also Causes Pol II Accumulation

**Figure 5:**
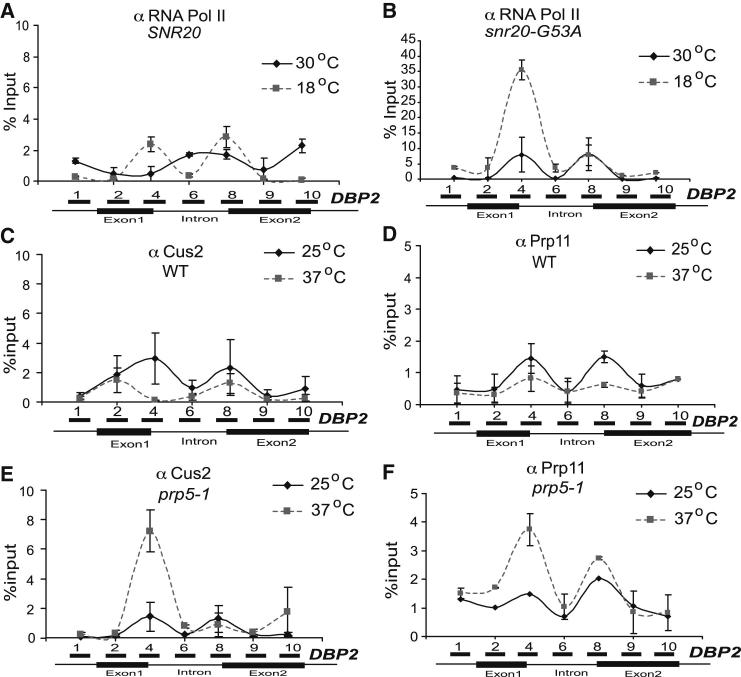
*snr20-G53A* Causes Pol II Accumulation over the Intron, and Cotranscriptional Recruitment of Cus2p Coincides with Pol II Accumulation in *prp5-1* Cells (A and B) Mid-log phase WT *SNR20* (A) and mutant *snr20-G53A* (B) cells grown at 30°C were shifted to 18°C for 1 hr. ChIP-qPCR (4F8 antibodies) shows the Pol II profile on *DBP2*. Solid and dashed lines denote 30°C and 18°C, respectively. Figure S5A shows a time course of the effect of the *snr20-G53A* mutation, as well as Pol II ChIP data for *ACT1*, *ASC1*, and *FMP27*. (C) WT cells containing TAP-tagged Cus2p were grown at 25°C and shifted to 37°C for 30 min. ChIP was performed using immunoglobulin G (IgG) sepharose followed by qPCR analysis on *DBP2*. (D) WT cells were treated and ChIP-qPCR was performed as for (C) but using rabbit anti-Prp11p instead of IgG. (E) *prp5-1* cells containing TAP-tagged Cus2p were treated and ChIP-qPCR was performed exactly as for (C). (F) *prp5-1* cells were treated and ChIP-qPCR was performed as for (C) but using rabbit anti-Prp11p instead of IgG. Figure S5 shows the background signal generated on the intronless *FMP27* gene. x axis indicates the position of amplicons generated during qPCR. Error bars indicate SE from two independent biological experiments, each assayed in duplicate by qPCR.

Next, we wanted to know whether the observed transcription defect is elicited by the defect in prespliceosome formation or by some other consequence of the *prp5-1* mutation. The *prp5-1* mutation causes accumulation of a complex with U2 snRNA stuck in the stem IIc form to which Cus2p is bound. Therefore, we tested the effect of the *snr20-G53A* mutation that hyperstabilizes the stem IIc configuration of U2 snRNA. This mutation causes a defect in prespliceosome formation at a low temperature, even in the presence of WT Prp5p (Wells et al., 1996; Zavanelli and Ares, 1991), and Cus2p remains associated with the stalled complex as it has higher affinity for the stem IIc form of U2 snRNA (Yan et al., 1998). ChIP analysis of WT and *snr20-G53A* cells showed accumulation of Pol II over the *DBP2*, *ACT1*, and *ASC1* introns in the *snr20-G53A* cells incubated 1 hr at 18°C (Figures 5A, 5B, and S5) and to an even higher level than the Pol II accumulation observed in *prp5-1* cells at elevated temperature. Therefore, Pol II accumulation occurred when prespliceosome formation was inhibited as a consequence of blocking the conversion of stem IIc to stem IIa in the U2 snRNA by two distinct mechanisms. The effect of *cus2Δ* on Pol II accumulation in the *snr20-G53A* mutant strain could not be tested, as the double mutant is inviable (Yan et al., 1998).

### Cus2p and U2 snRNP Also Accumulate over the Intron in *prp5-1* Cells

As the absence of Cus2p abolished Pol II stalling, we investigated whether there is any correlation between Pol II accumulation and the cotranscriptional recruitment of Cus2p or of the U2 snRNP in *prp5-1* cells. Prp11p is a U2 snRNP component that interacts with Cus2p (Yan et al., 1998). By ChIP analysis, both Cus2p and Prp11p were detected near the 5′ and 3′ ends of the *DBP2* intron in WT cells, with the signal being slightly higher at 25°C than 37°C in both cases (Figures 5C and 5D). The cotranscriptional recruitment pattern for U2 snRNP on *DBP2* is similar to an earlier report (Görnemann et al., 2005), except for a peak that is more 5′ detected here using an additional primer set. The dip in the signal observed between the 5′ and 3′ ends of the intron could be due to protein epitopes being masked in the spliceosome or due to the existence of distinct complexes. As a control, intronless gene *FMP27* was analyzed, showing the background levels generated (Figures S5H and S5I). In *prp5-1* cells at the restrictive temperature, the accumulation of both of these proteins increased over the intron, similarly to Pol II (Figures 5E and 5F), which likely represents accumulation of splicing complexes with stalled Pol II.

### A Branchpoint Mutation Affects Pol II Accumulation

**Figure 6:**
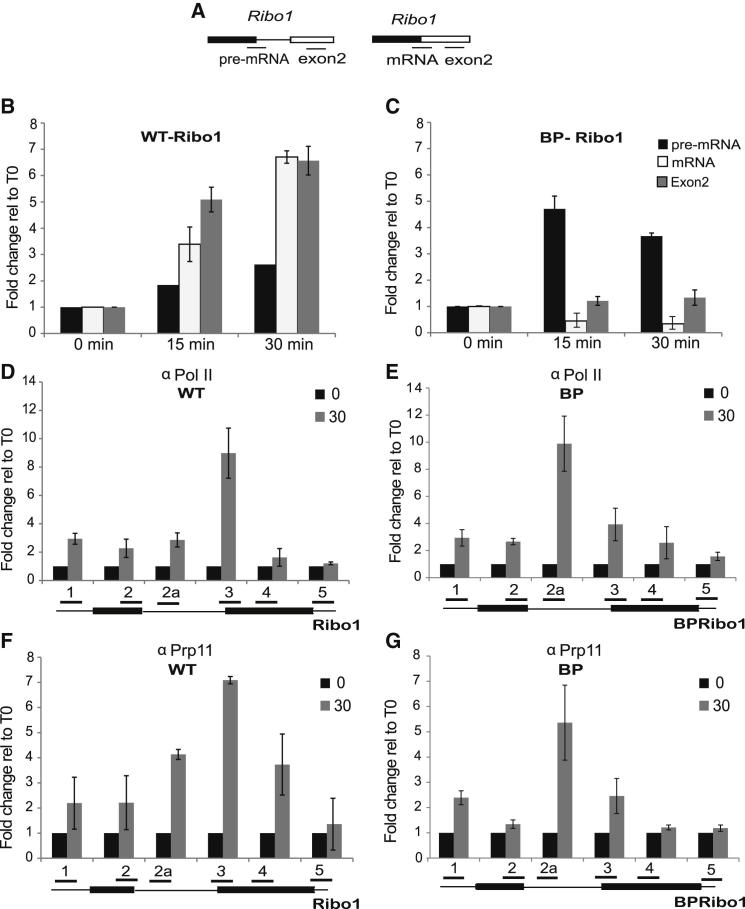
A Branchpoint Mutation Alters Pol II Accumulation on the Ribo1 Intron (A) Diagram indicating the qPCR amplicons used for RNA analysis. (B and C) RT-qPCR was performed on RNA from Ribo1 (WT) (B) and BPRibo1 (BP) (C) tetOFF cultures in which transcription was repressed by the presence of doxycycline (dox; T0) or after 15 or 30 min induction in the absence of dox. Pre-mRNA, mRNA, and exon2 levels are presented relative to the uninduced levels (T0). (D and E) Pol II ChIP analysis for WT (D) and BP (E) reporters as above. (F and G) Prp11p ChIP with WT (F) and BP (G), showing the U2 snRNP recruitment profiles. The positions of PCR amplicons are shown below the graphs. The primers for amplicons 1, 2, 3, 4, and 5 are as described by Alexander et al. (2010a). For amplicon 2a, see the Supplemental Information.

As prespliceosome formation depends on U2 association with the intron around the branchpoint, we tested the effect of a mutant branchpoint sequence. For this analysis we used the Ribo1 reporter gene with a T-to-A substitution at −2 relative to the branchpoint nucleotide (BPRibo1; Alexander et al., 2010a), which inhibits prespliceosome formation due to decreased complementarity with U2 snRNA. RT-qPCR analysis following induction of BPRibo1 showed pre-mRNA accumulation, no detectable mRNA production, and only low accumulation of exon2 compared to WT Ribo1 (Figures 6A–6C). ChIP analysis of the WT Ribo1 gene 30 min after induction showed a peak of Pol II near the 3′ end of the intron (Figure 6D), where splicing-dependent Pol II pausing was previously demonstrated (Alexander et al., 2010a). In the case of BPRibo1, accumulation of Pol II was observed more 5′ on the intron (Figure 6E). ChIP of the U2 snRNP protein, Prp11p, detected its cotranscriptional recruitment maximally near the 3′ end of the Ribo1 intron, but closer to the 5′ end of the BPRibo1 intron (Figures 6F and 6G), coinciding with Pol II accumulation.

## Discussion

Although there is good evidence for functional interactions between splicing and transcription in yeast cells (Alexander et al., 2010a), the mechanism(s) by which these processes are coupled and the functional significance of coupling remain unknown. We present several lines of evidence, based on analyses of the heat-sensitive *prp5-1* mutation, the cold-sensitive *snr20-G53A* mutation, and a branchpoint mutation in a reporter gene, showing that defects in the cotranscriptional formation of prespliceosomes trigger transcriptional elongation defects. In the case of the *prp5-1* mutation at the restrictive temperature, ChIP-qPCR analysis revealed accumulation of Pol II on intron-containing genes, and genome-wide ChIP-seq showed Pol II enriched on introns and depleted over downstream exons, compatible with a transcriptional elongation defect occurring over introns. This was supported by in vivo 4TU labeling of RNA that showed reduced nascent transcript levels for intron-containing genes. As deletion of *CUS2* suppressed the transcript production defect but not the splicing defect caused by *prp5-1*, we conclude that the reduced RNA accumulation in *prp5-1* cells at 37°C is Cus2p dependent and not simply a consequence of the splicing defect. The finding that the accumulated Pol II was strongly (compared to Pol II at the promoter) phosphorylated on Ser5, but not on Ser2, is consistent with a transcriptional elongation defect due to paused or stalled Pol II (Lin et al., 2008; Levine, 2011). However, our analyses do not rule out the possibility that there could be a checkpoint-induced rapid degradation of nascent transcripts.

Our analysis of ChIP-seq data for Pol II occupancy on intron-containing genes in WT cells revealed an elevated number of reads, indicative of higher Pol II occupancy around the 5′ ends of introns, which was enhanced in the *prp5-1* mutant at the restrictive temperature. As most yeast introns lie near the 5′ ends of genes, this peak of Pol II appears in the gene average plots (Figure 2) to extend from the transcription start site across the first exon and into the intron. However, in the case of genes with long first exons, such as *DBP2*, the ChIP-seq and ChIP-qPCR analyses show that the peak of Pol II at the 5′SS does not extend to the 5′ end of the gene. Furthermore, the peak of pSer5 observed by ChIP-PCR near the 5′ end of the *DBP2* intron is distinct from and much greater than the pSer5 signal at the 5′ end of the gene (Figures 1E and 1G) and so is likely due to new phosphorylation of Ser5 rather than failure of pSer5 to be dephosphorylated at promoter clearance. The very striking increase in ChIP-seq reads immediately downstream of introns in WT cells correlates well with splicing-dependent Pol II pausing that was observed to occur in this region of two reporter genes (Alexander et al., 2010a). For splicing-dependent effects on Pol II, spliceosome assembly must occur cotranscriptionally. Indeed, we found a correlation between introns that show enrichment >2-fold of Pol II over introns in the *prp5-1* mutant and introns that were previously reported to be highly cotranscriptionally spliced in WT cells (Carrillo Oesterreich et al., 2010).

We also found accumulation of Pol II over introns in *snr20-G53A* cells at 18°C (Figures 5A and 5B), a temperature at which this strain fails to form prespliceosomes (Zavanelli and Ares, 1991). Therefore, Pol II accumulation over introns occurred as a consequence of defective prespliceosome formation caused by defects in either of two different splicing factors.

The accumulation of Prp11p and Cus2p with a pattern similar to Pol II on the *DBP2* gene in *prp5-1* cells at 37°C is compatible with the failure of mutant Prp5p to release Cus2p from the cotranscriptionally assembled prespliceosome complex that accumulates in the region of the stalled Pol II (compare Figures 3D, 5E, and 5F). However, in the *prp5-1* strain, both Cus2p and Prp11p were detected further upstream on the intron than expected (Görnemann et al., 2005). In *S. pombe* and *S. cerevisiae*, Prp5p forms bridging interactions between the U1 and U2 snRNPs and is detected from the time of U1 recruitment to pre-mRNA (Xu et al., 2004; Kosowski et al., 2009; Shao et al., 2012). Similarly, Dönmez et al. (2004) reported that U2 and U1 snRNPs are in close proximity and “bridge” the 5′ and 3′ ends of the intron already in the human E complex, prior to complex A (prespliceosome) formation. If Cus2p and the U2 snRNP (or at least Prp11p) initially associate through Prp5p with U1 snRNP at the 5′ end of the intron prior to formation of a stable interaction between U2 snRNP and the branchpoint, this could represent a transitional complex (Perriman and Ares, 2000; 2010) that accumulates in *prp5-1* cells at the restrictive temperature.

Base-pairing between U2 snRNA and the branchpoint region of introns is critical for prespliceosome formation. To investigate the effect of a pre-mRNA with a mutant BP that destabilizes interaction with U2, we used our Ribo1 reporter system (Alexander et al., 2010a). Whereas Pol II accumulated at the 3′ end of the WT Ribo1 intron, BPRibo1, which has a mutation close to the BP nucleotide, accumulated Pol II further upstream on the intron. The transient accumulation of Pol II near the 3′ end of the Ribo1 intron is splicing dependent and apparently occurs after the first step of splicing (Alexander et al., 2010a). As BPRibo1 transcripts are unable to undergo the first step of splicing, Pol II is not expected to pause at the 3′ end of this intron, so the Pol II accumulation more 5′ on BPRibo1 indicates a different pausing or stalling event. Prp11p was also cotranscriptionally recruited more 5′ with BPRibo1 than with WT Ribo1. This is again compatible with a transitional complex that accumulates because of a failure to form a stable interaction at the BP. Pol II accumulation on BPRibo1 may therefore occur as a consequence of the failure of a prespliceosome to form on nascent transcript emanating from a downstream polymerase.

Cus2p is the putative ortholog of human Tat-specific factor 1 (Tat-SF1), a general transcriptional elongation factor that affects both transcription (e.g., Zhou and Sharp, 1996) and alternative splicing (Miller et al., 2011). Tat-SF1 associates with components of the cellular transcription machinery, including Pol II, P-TEFb, DSIF/hSPT4/5, and Paf1 (Chen et al., 2009 and references therein) and with the components of U1 and U2 snRNPs (Fong and Zhou, 2001 and references therein). However, whether the dual functions of Tat-SF1 in transcription and splicing are coupled is unclear (Fong and Zhou, 2001; Miller et al., 2011). Cus2p shares 46% sequence identity with Tat-SF1, and both proteins contain two RNA recognition motifs (RRMs) and an acidic CTD (Yan et al., 1998). Both proteins interact with Prp11p/SF3a66, a conserved component of U2 snRNPs (Yan et al., 1998). Detection of Cus2p and Prp11p near the 5′ end of the intron is comparable with the detection of their homologs (Tat-SF1 and U2 snRNP components) at the 5′ end of an intron in mammals (Kameoka et al., 2004), which was proposed to show a link between elongating polymerase and splicing.

In higher eukaryotes, Pol II pausing has been observed to occur near the promoter regions of genes, referred to as promoter proximal pausing. Originally associated with stress response genes, it is now recognized to occur more widely, especially at developmentally regulated genes, and has been proposed as a mechanism for holding Pol II in a poised state, ready for rapid activation (reviewed in Levine, 2011; Li and Gilmour, 2011). Pol II pausing is characterized by pSer5 on the CTD, with phosphorylation of Ser2 being important for release from the paused state. Here, we describe another form of Pol II pausing, again characterized by the accumulation of Pol II with pSer5, but within the body of intron-containing genes, and associated with splicing. We propose that, in the *prp5-1* and *snr20-G53A* strains, release of the paused Pol II is inhibited due to the defect in prespliceosome formation.

**Figure 7:**
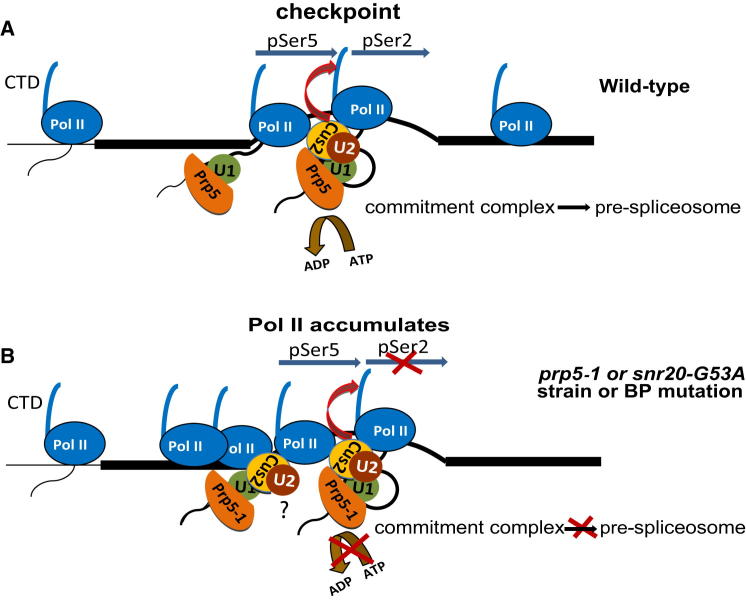
Model Illustrating the Proposed Splicing-Coupled Transcriptional Checkpoint Associated with Prespliceosome Formation Mediated through Cus2p (A) Cartoon depicting cotranscriptional recruitment of U1 (green circle), Prp5p (orange), U2 (brown), and associated Cus2p (yellow) to the nascent RNA linked to Pol II. Cus2p may act as a checkpoint factor, triggering the checkpoint and resulting in transient pausing of Pol II. With formation of the functional prespliceosome, Cus2p is displaced, the checkpoint is satisfied, and Pol II becomes phosphorylated on Ser2 of the CTD and is released. (B) The *prp5-1* strain assembles U1, U2, Prp5-1p, and Cus2p cotranscriptionally as in (A) but fails to promote prespliceosome formation and displacement of Cus2p. In this case, the checkpoint is not satisfied, and Pol II accumulates over the intron. The *snr20-G53A* and intron branchpoint mutations also trigger the checkpoint, although a role for Cus2p has not been demonstrated.

To explain all of our observations, we propose the existence of a splicing-dependent transcriptional checkpoint associated with prespliceosome formation (Figure 7). The reduced transcriptional elongation accompanied by accumulation of Pol II with pSer5 resembles the transcriptional checkpoint that is associated with capping of nascent transcripts (Orphanides and Reinberg, 2002), but the capping checkpoint occurs more upstream, close to the transcription start site. In the case of the *prp5-1* mutation, the presence of Cus2p is necessary to trigger our proposed splicing-dependent checkpoint, making Cus2p a candidate checkpoint factor. In a parsimonious model, transcriptional pausing may be triggered by recruitment of Cus2p along with the U2 snRNP to the assembling spliceosome. After remodeling of the U2 snRNA by Prp5p, dissociation of Cus2p from the complex might signal that the prespliceosome has been formed and the checkpoint is satisfied, releasing Pol II from pausing. However, in the *prp5-1* strain or the *snr20-G53A* strain at the restrictive temperature, the checkpoint is triggered, but not satisfied, as Cus2p remains in the complex, resulting in the accumulation of stalled Pol II that is strongly phosphorylated on Ser5. Release of paused Pol II at a checkpoint is predicted to require phosphorylation of Ser2 of the CTD. Therefore, Cus2p might potentially affect the function of the yeast CTD kinase complexes, BUR and CTK. Intriguingly, Tat-SF1 interacts with the mammalian CTD kinase P-TEFb. However, a recent investigation failed to find evidence for interaction between *CUS2* and mutations in *BUR2*, *CTK2*, *DST1*, or genes encoding other transcription elongation factors (McKay and Johnson, 2011).

We previously proposed that transient, splicing-dependent Pol II pausing near intron 3′ ends might indicate the presence of a splicing-coupled transcriptional checkpoint associated with the second step of splicing (Alexander et al., 2010a). This would depend on the splicing reaction completing cotranscriptionally, and indeed, the Ribo1 transcripts were shown to be ∼90% cotranscriptionally spliced, as measured by production of spliced transcripts prior to their 3′ end cleavage and polyadenylation (Alexander et al., 2010b; Aitken et al., 2011). The Pol II accumulation observed here in response to a defect in prespliceosome assembly appears to be a different event. It is conceivable that Pol II pausing may occur repeatedly on intron-containing genes if multiple transcriptional checkpoints exist that respond to different stages in the splicing process.

We anticipate that, under normal splicing conditions, Pol II pausing at checkpoints is highly transient and that the ability to detect it, as well as the location of the pausing event on the gene, could be gene specific if it depends on the rate of transition through the checkpoint-coupled splicing event. This would, in turn, depend on many factors, including the kinetics of cotranscriptional recruitment of different splicing factors to the intron. It is anticipated that splicing-coupled transcriptional checkpoints could be affected by and might also affect splice-site selection.

Pol II that is strongly phosphorylated on Ser5 of the CTD has been observed to accumulate on alternatively spliced exons under circumstances of exon inclusion in mammalian cells (Batsché et al., 2006). Considering that the rate of transcriptional elongation can affect alternative exon inclusion (reviewed in Kornblihtt et al., 2013), Pol II pausing in a transcriptional checkpoint might contribute to the regulation of alternative splicing by controlling the progress of transcription while decisions are made about alternative splicing events.

## Experimental Procedures

### RNA Isolation and RT-qPCR

RNA extraction, RT-qPCR, and ChIP-qPCR were as described (Alexander et al., 2010a). In vivo 4TU labeling of RNA, yeast strains, and oligos used as primers are described in Supplemental Experimental Procedures.

### ChIP-Seq Analysis

ChIP DNA was extracted from *prp5-1* cells grown at 25°C and 37°C and WT cells at 25°C. Library was prepared adapting the Illumina protocol (Schmidt et al., 2009). Library DNA was gel eluted, purified, and quantified by Bioanalyzer and qPCR. Solexa sequencing, base calling, and quality control were carried out in the GenePool Genomics Facility at the University of Edinburgh. Single-end reads (with 50 bases) were adaptor clipped, quality trimmed, and filtered and then mapped to the *S. cerevisiae* sacCer3 genome assembly using bwa version 0.6.1. Uniquely mapped reads with mapping quality ≥ 20 were selected for further analysis. Samples were normalized by number of mapped reads in order to account for variable sequencing depth. Subsequent analyses to define polymerase distribution were carried out using R and perl scripts interfaced with a Galaxy server. The scripts are available at https://github.com/swebb1/ktc_paper_2013. For further details see Supplemental Experimental Procedures.

## Accession Numbers

The sequence data have been deposited in NCBI’s Gene Expression Omnibus (Edgar et al., 2002) and are accessible under accession number GSE53647.

## Supplemental Information

Document S1. Supplemental Experimental Procedures and Figures S1–S5

Table S1. Details of Peak Enrichment Analysis on Intron-Containing Genes, and the List of Genes Used for Proximity Plot Analysis, Related to Figures 2 and S2 Sheet 1 lists the genes that show fold change > 2 at the restrictive temperature in *prp5-1* used for average plot analysis (Figure S2B–S2D), the regions showing peak enrichment, and their proximity to different features (TSS, 5′SS, 3′SS, BP, and 3′end). Chromosome coordinates and length in bases between gene features, including exon2 lengths, are given in separate columns. Other data sets used for correlations are also shown in separate columns, as indicated. Other sheets provide the lists of intron-containing genes used for proximity plot analyses (Figures 2C and 2D).
