# Supplementary material for: A Splicing-Dependent Transcriptional Checkpoint Associated with Prespliceosome Formation
Source: Mol Cell. 2014 Mar 6;53(5):779–90. doi: 10.1016/j.molcel.2014.01.017 (PMC3988880; doi:10.1016/j.molcel.2014.01.017)
Supplement: Document S1. Supplemental Experimental Procedures and Figures S1–S5 [file mmc1.pdf]

Molecular Cell, Volume *53*

Supplemental Information

A Splicing-Dependent Transcriptional Checkpoint Associated with Prespliceosome Formation

Keerthi T. Chathoth, J. David Barrass, Shaun Webb, and Jean D. Beggs

SUPPLEMENTAL FIGURES

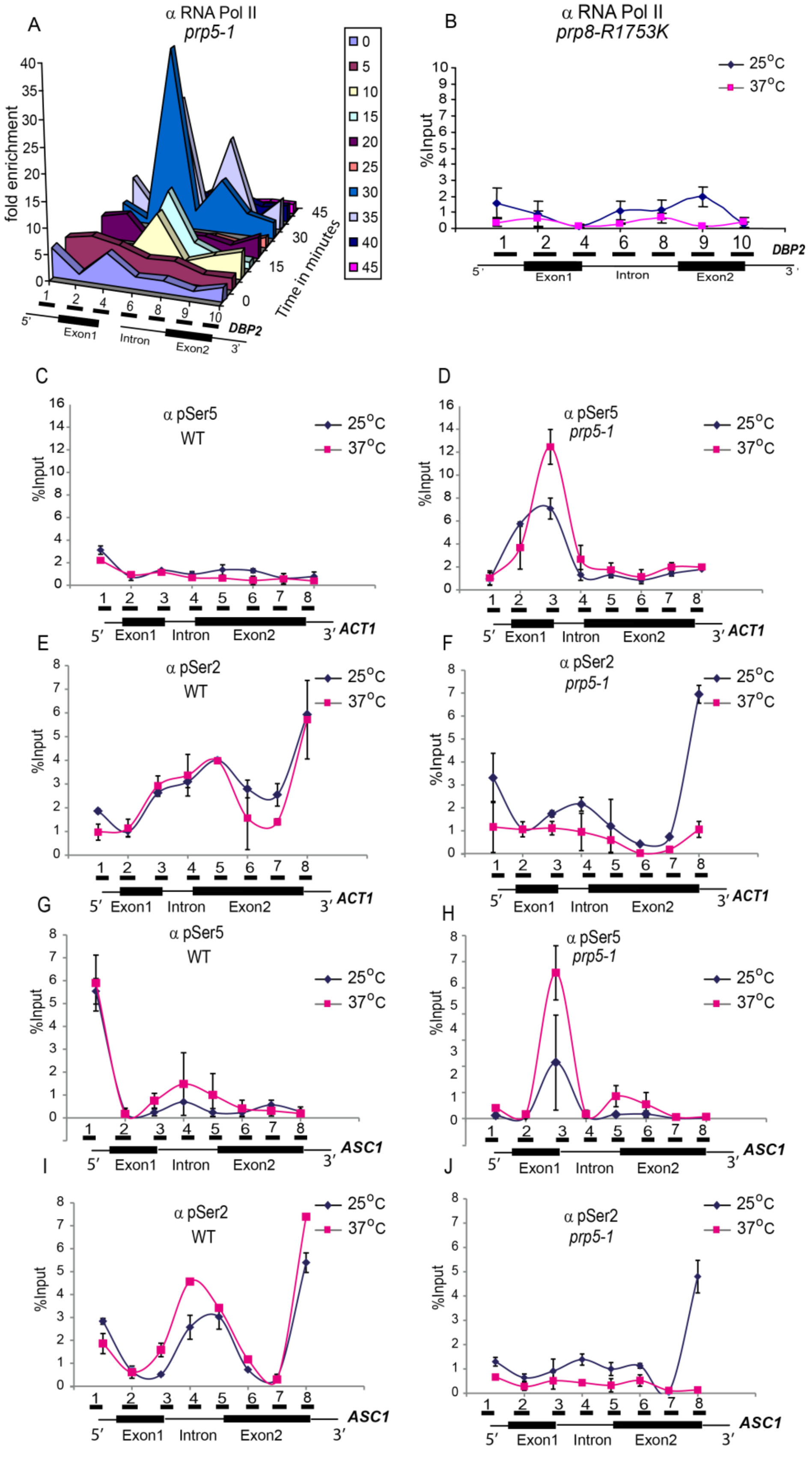

Figure S1. ChIP analysis of Pol II in *prp5-1* cells at 37°C. Related to Figure 1.
(A) For kinetic ChIP analysis of Pol II, WT and *prp5-1* cells were shifted from 25°C to 37°C, samples were formaldehyde crosslinked at 5 minute intervals and ChIP was performed for *DBP2*, using 4F8 antibody. Results are presented as fold over background (no antibody). X-axis: position of amplicons. Z-axis: time (min) at 37°C. Based on this, 30 min was chosen as the incubation time at 37°C in subsequent experiments. (B) RNA Pol II ChIP on *DBP2* gene in *prp8-R1753K* using same antibody and methods as described for Figure 1. (C-J) WT and *prp5-1* cultures were treated as described for Figure 1 and ChIP-qPCR was performed for *ACT1* and *ASC1* using 3E8 (pSer5) or 3E10 (pSer2) antibodies. See Alexander et al (2010a) and Table below for oligo primer details.

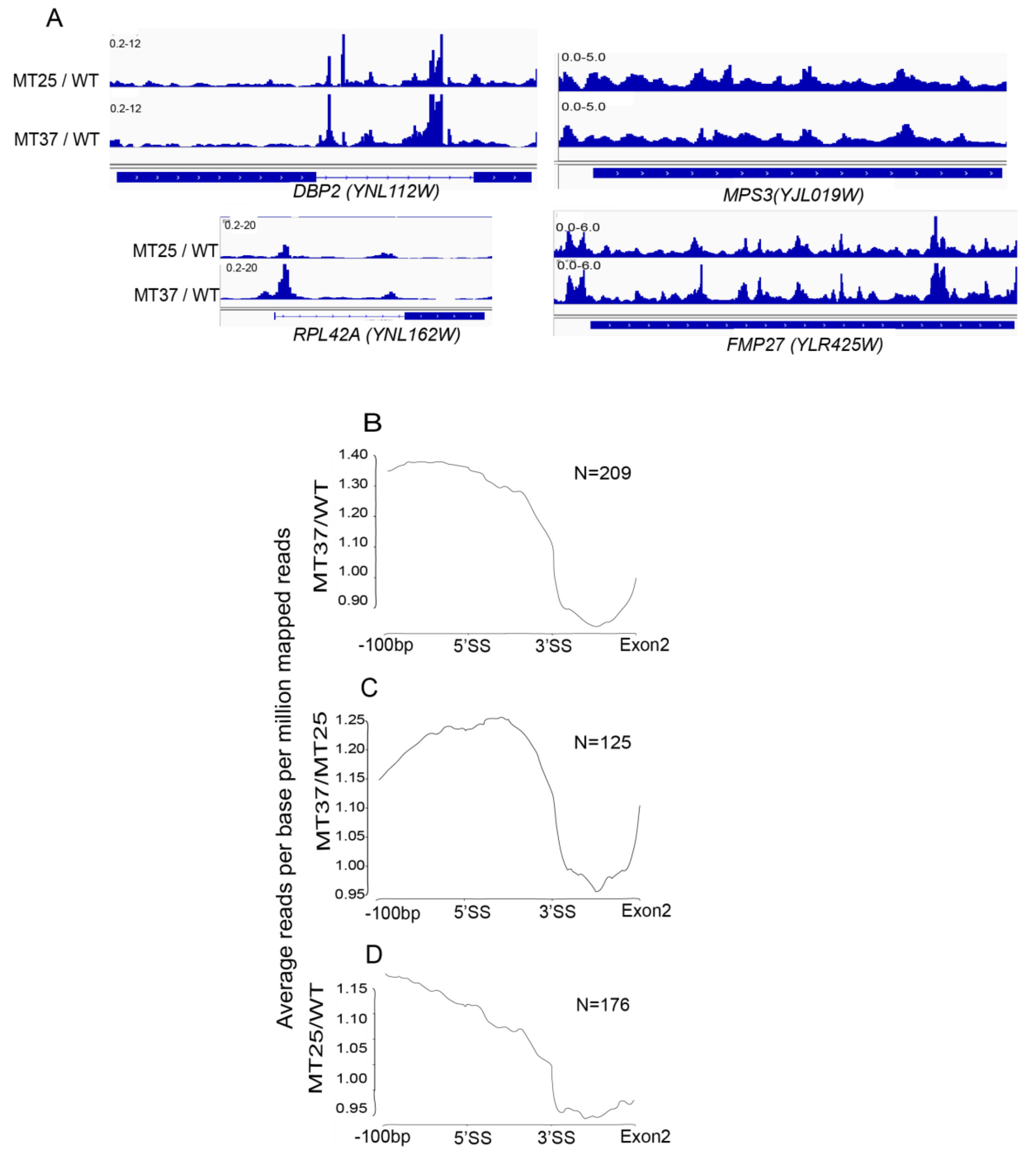

Figure S2. Pol II accumulates over introns in *prp5-1* cells. Related to Figure 2.
(A) Pol II ChIP-Seq analysis in *prp5-1* (MT) and WT cells on individual genes. Mapped and aligned reads were normalised to reads per million and presented as MT at 37°C/WT or 25°C/WT. IGV (Integrative Genomics Viewer) displays peaks of Pol II enriched over introns near 5' end and close to BP on intron-containing *DBP2* (2643bp) and *RPL42A* (833bp*)*, and intronless *MPS3* (2049bp) and *FMP27* (7887 bp). Y-axis: relative fold enrichment. X-axis: positions of exons. (B-D) Averaged Pol II enrichment profiles for all intron-containing genes that show >2-fold enrichment in *prp5-1* cells at 37°C or 25°C compared to WT (MT37/WT, MT25/WT), and mutant at 37°C compared to 25°C (MT37/MT25). Genes were divided into three equal bins representing 100 bp upstream of the 5'SS, the intron, and 3'SS to the stop codon, as indicated on the X-axis. Y-axis shows the reads per base per million mapped reads.

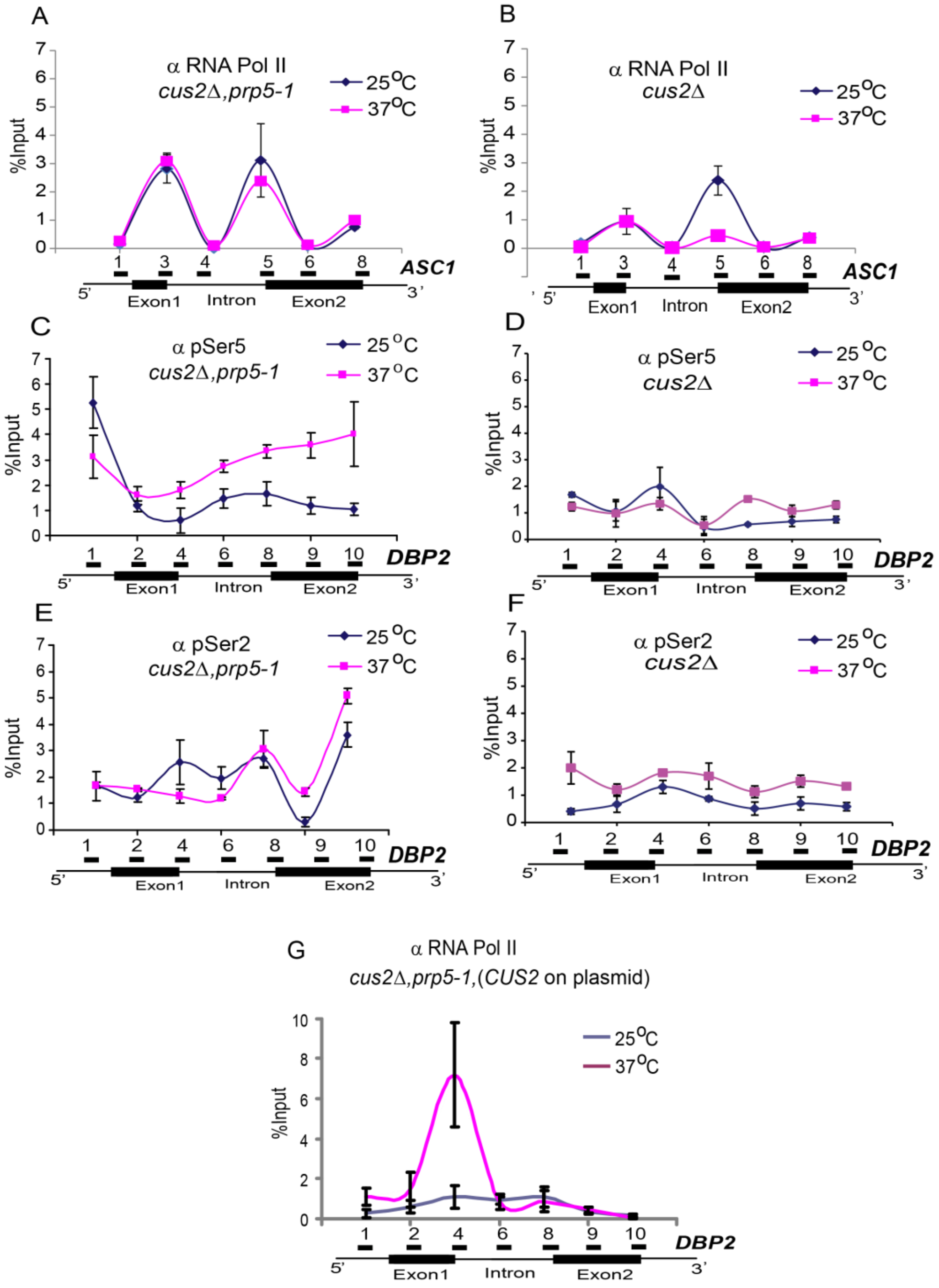

Figure S3. Cus2p is required for Pol II accumulation in *prp5-1* cells. Related to Figure 3. (A-F) ChIP-qPCR performed with the same crosslinked samples from *cus2*Δ,*prp5-1* and *cus2*Δ cells as for Figures 3B and 3C, using antibodies 4F8 for *ASC1*, and 3E8 (pSer5) or 3E10 (pSer2) for *DBP2*, as indicated. (G) *cus2*Δ, *prp5-1* cells were transformed with plasmid-encoded *CUS2* and Pol II ChIP was performed at 25°C (blue) and 37°C (red) followed by qPCR analysis on *DBP2*, using 4F8 antibodies.

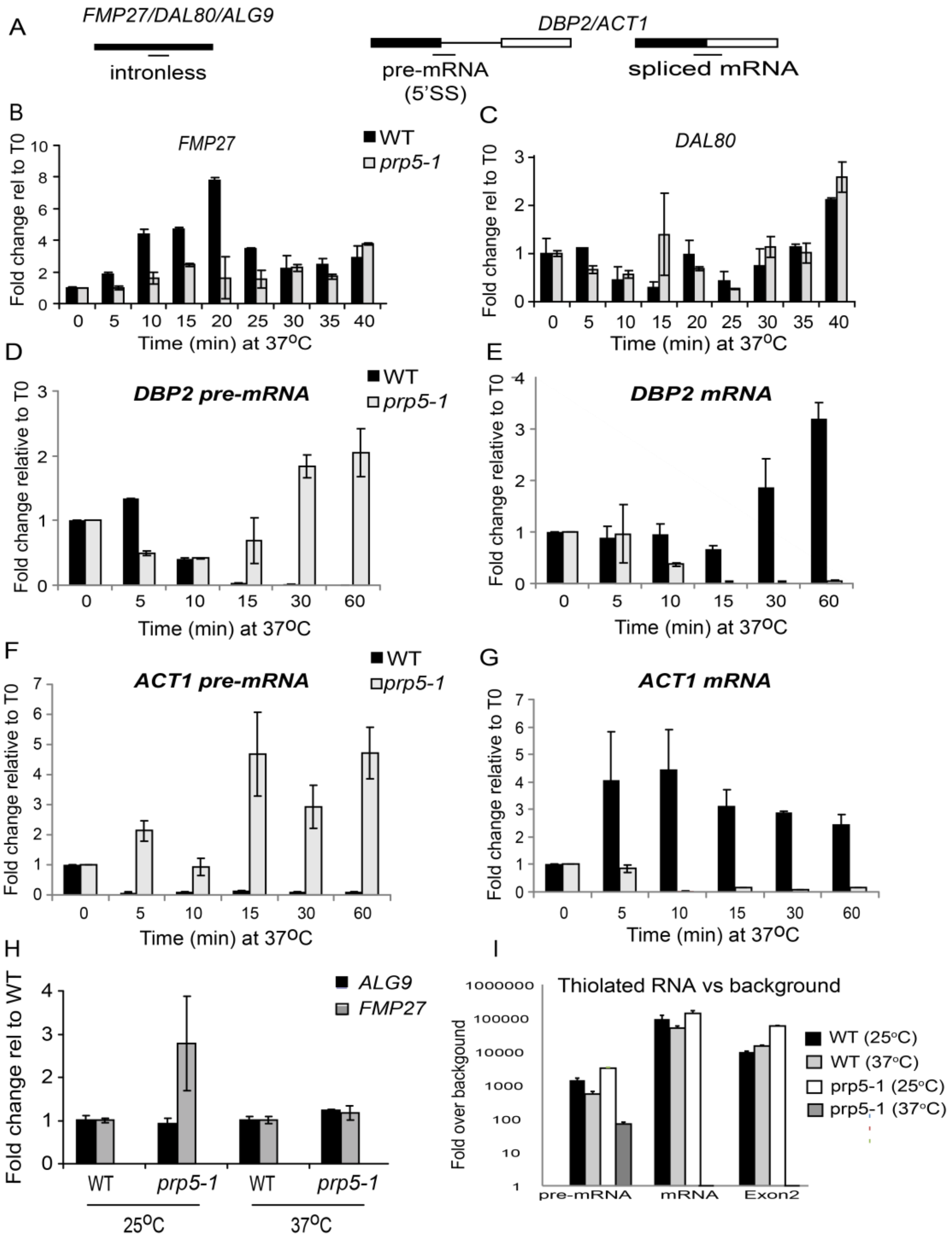

Figure S4. Splicing defect in *prp5-1* cells at 37°C. Related to Figure 4.

(A) Diagrams indicating the positions of amplicons generated by qPCR. (B-G) RT-PCR analyses using the same RNA as used for Figures 4B and 4C, (B-C) intronless genes *FMP27* and *DAL80*, (D-G) pre-mRNA and spliced mRNA of intron-containing genes *DBP2* and *ACT1*. This shows the relative accumulation of pre-mRNAs and reduction of mRNAs in mutant cells. Black bar, wild-type; gray bar, *prp5-1*. (H) Intronless genes *ALG9* and *FMP27*, assayed using the same 4TU-labelled RNA as for Figure 4D. (I) As a measure of the effectiveness of the affinity purification of nascent RNA away from pre-existing RNA, the values for *DBP2* pre-mRNA, mRNA and exon2 are presented as fold over background (the same analysis without 4TU), with standard error shown. N.B. mRNA and exon2 for *prp5-1* at 37°C were at background level.

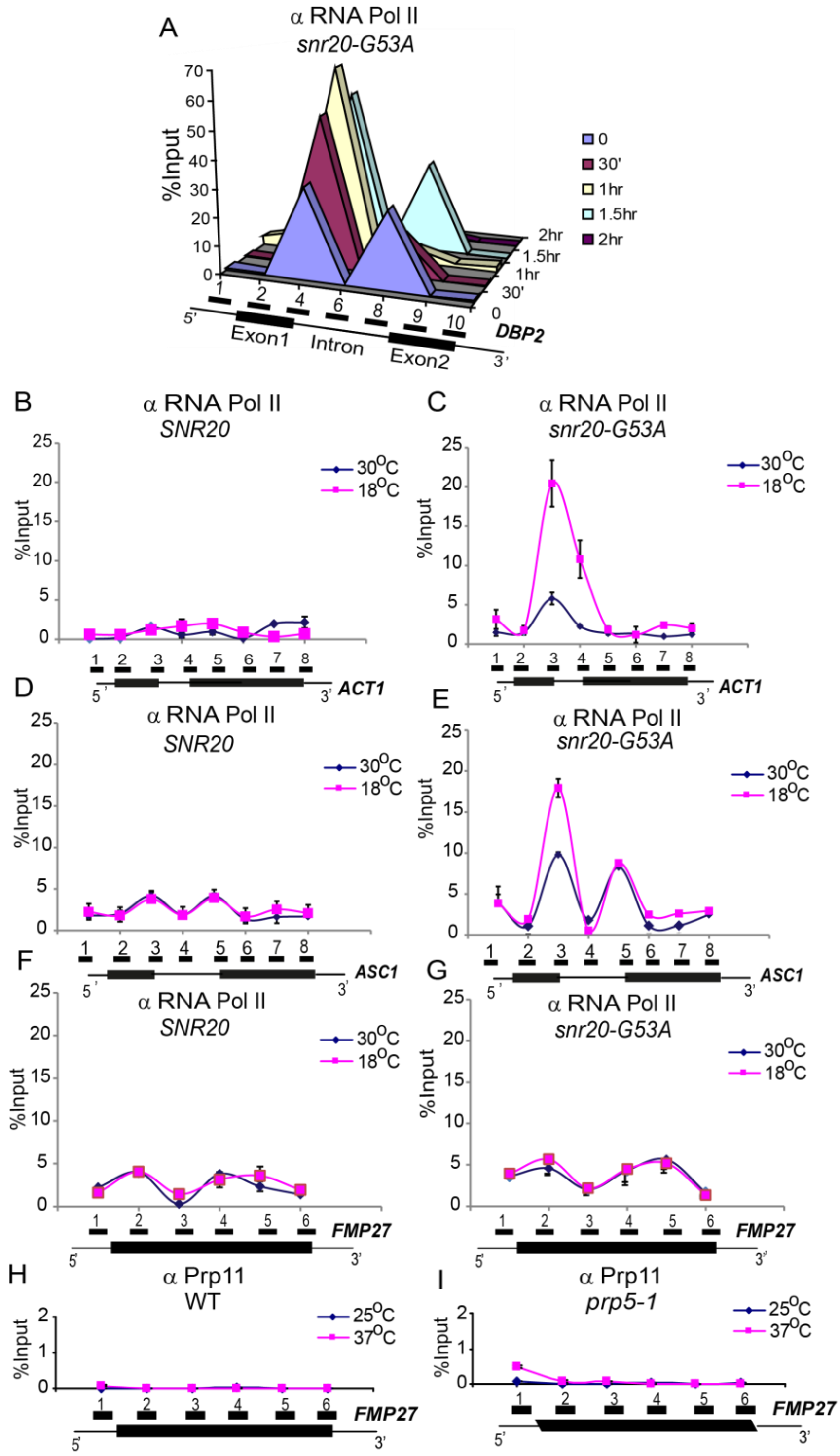

Figure S5. Pol II ChIP analysis in *snr20-G53A* mutant cells at 18°C. Related to Figure 5. (A) *snr20-G53A* mutant cells were temperature shifted as described for Figure 5. Samples were collected at 30 min intervals, formaldehyde crosslinked and Pol II ChIP-qPCR analysis was performed on *DBP2*. (B-G) ChIP qPCR with the same ChIP samples from WT *SNR20* and mutant *snr20-G53A* cells as described in Figure 5; Pol II profile on intron-containing genes *ACT1* and *ASC1* and intronless gene *FMP27*. (H-I) Prp11p ChIP-qPCR analysis on intronless gene *FMP27*, using the same ChIP samples as for Figures 5D and F, showing the background level of signal.

Table S1. Details of peak enrichment analysis on intron-containing genes and the list of genes used for proximity plot analysis. (Separate Excel file) Related to Figures 2 and S2.

Sheet 1 lists the genes that show >2-fold change at the restrictive temperature in *prp5-1* used for average plot analysis (Figure S2B-D), the regions showing peak enrichment, and their proximity to different features (TSS, 5'SS, 3'SS, BP and 3'end). Chromosome coordinates, length in bases between gene features, including exon2 lengths, are given in separate columns. Other datasets used for correlations are also shown in separate columns as indicated. Other sheets provide the lists of intron-containing genes used for proximity plot analyses (Figures 2C and 2D).

SUPPLEMENTAL EXPERIMENTAL PROCEDURES

Table of yeast strains used

SPJKC5.41	*MATa leu2 his ura 3-52 prp5-1 cus2::KAN*	This study
SPJ5.41	*MATa leu2 his ura 3-52 prp5-1*	Beggs lab
SPJ5.041	*MATa leu2 his ura 3-52*	Beggs lab
SPJKC5.09	*MATa leu2 his ura 3-52 cus2::KANR*	This study
DS4D (WT *SNR20*)	*MATa prp5::KanMx snr20::HIS3 cus2::KanMx trp1 ura3 leu2 lys2pIP45 (Prp5+U2 on URA)*	Perriman et al., 2003
DS4D (*snr20-G53A*)	*MATa prp5::KanMx snr20::HIS3 cus2::KanMx trp1 ura3 leu2 lys2pIP45 (PRS314GPD Prp5 + U2-G53A LEU2)*	Perriman et al., 2003
YRA1	tetOFF strain YIK120 plus *his3::Ribo1-Nat*	Alexander et al., 2010b
YRA1-BP	tetOFF strain YIK120 plus *his3::BPRibo1-Nat*	Alexander et al., 2010b

Primers used for RTqPCR

DBP2 premRNA F	AAAATTGACTTTAATTAGTCGTTTTGAGAGACGGG
DBP2 premRNA R	GACATGAAGTGCAAATCATGAACACAAATACACTT
DBP2 intr-ex2 F	AACATGAATTTGTGGGGGGCATGAAAATA
DBP2 intr-ex2 R	CCTGGCATATCGTAGTTGATAACGTAATTGATACC
DBP2 mRNA F	GATGTGGCCGCCAGAGGTATC
DBP2 mRNA R	TGCTCTACCAGTTCTACCGATTCTGTG
APE2 exon2 F	CGGTCTGGTTGCTGATGTCAAG
APE2 exon2 R	TTTGGTCCCAGACGACAAATGA
ACT1 exon2 F	TCTGCCGGTATTGACCAAACTACTTA
ACT1 exon2 R	CCGGACATAACGATGTTACCGTATAA
FMP27 F	GGGGTCACGATCATTTTGGAAG
FMP27 R	CATCAGCAATGATGGTCTGGC
DAL80 F	AAGTTGCACGGGGAACCAAG
DAL80 R	TGGGTCGTTAGAATGGGTATTGG

Primers used for ChIP qPCR

DBP2 -20 F (1)	TAAGGCAAATTTAGAGCAAATATGACTTACGGTGG
DBP2 +92 R (1)	TTTCTATCAGAGTTTCTTCCACCGCGGA
DBP2 +431 F (2)	GTTGGCCAATGGCTTTATCT
DBP2 +524 R (2)	CCAATAATGGTTGAGCGTTG
DBP2 +750 F (3)	GTTCGCGACAGATCGGACAGTGAGATT
DBP2 +831 R (3)	GCTTTGGAATATCGTGTCCGGAAATAGTCAT
DBP2 +1225 F (4)	GGTAGATCCCCAATTATGGTTGCTACTGATGTG
DBP2 +1357 R (4)	AGCCATTAGGAAATAGCTGTAATATCGTTAGGGGT
DBP2+1506 F (5)	ATTCTCGATGGGGAGTATCG
DBP2+1567 R (5)	CATCCTCCCAATATTAGTTTTCTG
DBP2 +1594 F (6)	AAAATTGACTTTAATTAGTCGTTTTGAGAGACGGG
DBP2 +1680 R (6)	GACATGAAGTGCAAATCATGAACACAAATACACTT
DBP2 +1705 F (7)	GGCGCAAATAAACAAGGAAT
DBP2 +1781 R (7)	ACAAATGCCAAGCATTGAAA
DBP2 +2156 F (8)	AACATGAATTTGTGGGGGGCATGAAAATA
DBP2 +2385 R (8)	CCTGGCATATCGTAGTTGATAACGTAATTGATACC
DBP2 +2447 F (9)	TGAAATACGACAGGAGATCTTATGGTGGCG
DBP2 +2690 R (9)	TCTCTGCCTGTTACCGCCGTAACCA
DBP2 +2867 F (10)	TAGGACAGACACTTTTCTTTGTTCTCGTACAACCC
DBP2 +3143 R (10)	CCATAAGCGCTAGTGCACGACTTCTTTGTAAGT
Asc1 -241 F (1)	GTGCTTCTCCAGCGAAAGTC
Asc1 -132 R (1)	AAAGGAATAGCCCAATGCCAAA
Asc1 -15 F (2)	TAAATAAAGTGAAAAAATGGCATCTAACGAA
Asc1 +98 R (2)	AATAGGTTTGGTTGACCAGCAGAAGTAG
Asc1 +459 (5SS) F (3)	AGTCAGAGTTGTTCCAAACGAAAAAGCTGATGATG
Asc1 +605 (5SS) R (3)	AAATCCACTTTTCTTCTTCTACTCGATTGTCATCA
Asc1 +600 F (4)	GATTTGTGTATGCCATTCAAATGATGT
Asc1 +692 R (4)	TTGGTGATAATTGGTATGTCTCATTCG
Asc1 +719 (3SS) F (5)	CTCTGCTCTTCTCTTTACTCGTTATGTCAAAATGG
Asc1 +850 (3SS) R (5)	TGAAGTCAGCTTCAATTTGGAATTGGTTTAAGTT
Asc1 +897 F (6)	ACTTTGATTGCTTCCGCTGGTAA
Asc1 +988 R (6)	CATCTTGGGCAGACAAAGTG
Asc1 +1150 F (7)	CTTGGTCTGCTGACGGTCAAAC
Asc1 +1220 R (7)	ATAACTTGCCAAACTCTAATGACG
Asc1 +1341 F (8)	TCGTCATAGATTTCGAAGTAATGAAAGAAA
Asc1 +1417 R (8)	TTTCGCAGCAAACAGAAAGCA
Act1 -533 F (1)	CCACAGCAATTAATGCACAACATTTA
Act1 -449 R (1)	GGCATATGTTTTTAAGGGTTTTGAGG
Act1 -139 F (2)	TTCCCCTTTCTACTCAAACCAAGAAG
Act1 -43 R (2)	AAGCGTGAAAAATCTAAAAGCTGATG
Act1 -71 (5SS) F (3)	TACATCAGCTTTTAGATTTTTCACGCTTACTGCTT
Act1 +34 (5SS) R (3)	GATGGTGCAAGCGCTAGAACATACCAGAAT
Act1 +368 (3SS) F (4)	TGTACTAACATCGATTGCTTCATTCTTTTTGTTGC
Act1 +413 (3SS) R (4)	GACGATAGATGGGAAGACAGCACGAGGA
Act1 +561 F (5)	ATCTGGCATCATACCTTCTACAACGA
Act1 +653 R (5)	GTTTGATTTAGGGTTCATTGGAGCTT
Act1 +1119 F (6)	TCTGCCGGTATTGACCAAACTACTTA
Act1 +1210 R (6)	CCGGACATAACGATGTTACCGTATAA
Act1 +1595 F (7)	ATGTGTTTTGTCTCTCCCTTTTCTACGAAAATTTC
Act1 +1620 F (7)	CGAAAATTTCAAAAATTGACCAAAAA
Act1 +1727 R (8)	ATGATACACGGTCCAATGGATAAACA

Act1 +1734 R (8)	TGATCATATGATACACGGTCCAATGGATAAACAT

FMP27 -44 F (1)	GAACATAAGAATCCTTAGAAAAGCCCTTTACCTCG
FMP27 +63 R (1)	CCATAAGAAAGTCACTGCAAATATAAGCCACTTGT
FMP27 +474 F (2)	AAGATTTGATTCCTTTTTGAGAAAACTTCTTTGGA
FMP27 +593 R (2)	CCATCCTTCAGAGGATTCATAATTTCACCAATT
FMP27 +1051 F (3)	TTGAATCTAAATCGAAAACATCAAAGCCACG
FMP27 +1154 R (3)	AATTTTTGAGAGAACAAATTGGTTTCGCCA
FMP27 +4525 F (4)	AGACCTAGTACCAATACAATGTTCATTCCAAACCA
FMP27 +4642 R (4)	CCTTGTCTGCTTTTTCGTTTTTACTTGATGTAGTG
FMP27 +7589 F (5)	TGAACAGCTTCAAACTTTGTATCAGTTATAAGGGC
FMP27 +7673 R (5)	GGGAAATTGAAAACAAAGTTAGTAACGTTAGCCAA
FMP27 +8058 F (6)	AAAGTAAAAAAAATAATGGTCTCTAGCGGGATCG
FMP27 +8137 R (6)	CCCAGTTGGTTAAGGCACCGTGCTAATAAC

RNA isolation and RT-QPCR

RNA was isolated from yeast cells using GTC phenol (Sambrook et al., 1989), except for 4TU labelled RNA, where hot phenol was used (Schmitt et al., 1990). RT-qPCR was as described previously (Alexander et al., 2010a), using oligos listed above.

Temperature shift experiments

Heat-sensitive mutants were grown at 25°C to the required OD, and then an equal volume of warmed growth medium was added to shift immediately to the restrictive temperature (37°C). Cold-sensitive mutants were grown at 30°C and shifted to 18°C by adding an equal volume of growth medium at 4°C. Cells harvested just before the shift were taken as control for the permissive temperature.

In vivo RNA labelling with 4-thiouracil

Cells containing plasmid pAT1 with the uridine permease *FUI1* gene were grown in SD -Ura –Leu to OD600 = 0.5 to 0.7 at 25°C or after 30 minute shift to 37°C, then 4TU was added to 0.5 mM final concentration. After 2 minutes, cells were fixed by addition of an equal volume of methanol on dry ice and pelleted at 4°C. RNA, isolated by the hot phenol method, was incubated with EZ-link HPDP-biotin (Thermo Scientific) to final concentration 0.8 mM at 65°C for 3 hrs. 4TU-labelled RNA was affinity purified as described previously (Swiatkowska et al., 2012).

Chromatin immunoprecipitation and qPCR

Cells were crosslinked with formaldehyde according to Alexander et al. (2010a), spheroplasted and MNase digested according to Furuyama and Biggins (2007) except that SOB (1M Sorbitol, 50mM Tris-Cl (pH 7.5), 0.5mM CaCl2 and 10mM β-mercaptoethanol) was used instead of SPC. Enzymatic reaction was stopped by the addition of 10mM EDTA and the supernates containing chromatin fragments were used for ChIP analysis. ChIP was performed as described (Alexander et al., 2010a) using antibodies 4F8, 3E10 and 3E8 (HemholtzZentrum Mũnchen). Primers are listed in the table above. Experiments were performed at least in biological duplicate, sometimes in triplicate, as mentioned in figure legends, with each sample assayed by qPCR in duplicate.

ChIP-Seq data analysis

To identify regions of enrichment in mutant over wild type, the average number of normalized reads per base was calculated for each sample in a 10 base window across the genome. A mutant to wild-type ratio was applied to each window and set to 1 if both values were low in order to remove bias due to small fluctuations at low read depth. Enriched coverage of genome features (exon1, exon2, intron, intronless genes) was calculated as the percentage of windows overlapping a feature that exceeded the 2-fold enrichment threshold. Regions of interest in the mutant were defined by clustering windows, with a ratio greater than a 2-fold enrichment threshold, within 50 bp of each other. Clusters contain at least 3 such windows. Candidate genes were identified by mapping regions of interest to intron-containing gene units annotated by the Saccharomyces Gene Database (SGD) (329 genes). This list was further filtered for non-mitochondrial genes. Proximity plots were created by extending the features of interest (TSS, 5’SS, branch point, 3’SS, 3’end) by 500 bases either side or splitting this region into windows of 10 bases. TSS and 3’end annotations were downloaded from the SGD website:
(http://downloads.yeastgenome.org/published_datasets/Yassour_2009_PMID_19208812/).
An average profile was generated by taking the mean normalised value for every window and relative profiles are the result of plotting log2 fold change between two samples. The significance of each peak was determined by applying a paired Wilcoxon Signed Rank test (Mann Whitney U) using normalised mean values from all 10 bp windows within the peak region to compare two populations (MT37/WT). The peak regions are as follows: -100 to +100 relative to TSS, 5'SS and 3'end, -200 to 0 relative to BP and 0 to 300 relative to 3'SS.

Ribo1 induction

Cultures of tetOFF Ribo1 or tetOFF BPRibo1 were grown in YPDA at 30°C to log phase in the presence of doxycycline (4ug/ml). Cells were pelleted, washed and resuspended in medium without doxycycline, then grown for 30 min to induce the reporter genes. Samples were collected immediately before the shift (0) or 30 min after (30).

SUPPLEMENTAL REFERENCES

Furuyama, S., and Biggins, S. (2007). Centromere identity is specified by a single centromeric nucleosome in budding yeast. Proc. Natl. Acad. Sci. U.S.A. *104*, 14706–14711.

Sambrook, J., Fritsch, E. F., and Maniatis, T. (1989).Molecular Cloning: A Laboratory Manual, 2nd ed. (Cold Spring Laboratory, Cold Spring Harbor, NY)

Schmidt, D., Wilson, M.D., Spyrou, C., Brown, G.D., Hadfield, J., and Odom, D.T. (2009). ChIP-seq: using high-throughput sequencing to discover protein-DNA interactions. Methods *48*, 240–248.

Swiatkowska, A., Wlotzka, W., Tuck, A., Barrass, J.D., Beggs, J.D., and Tollervey, D. (2012). Kinetic analysis of pre-ribosome structure in vivo. RNA New York N *18*, 2187–2200.
